# Cohort profile: characterisation, determinants, mechanisms and consequences of the long-term effects of COVID-19 – providing the evidence base for health care services (CONVALESCENCE) in the UK

**DOI:** 10.1136/bmjopen-2024-094760

**Published:** 2025-06-05

**Authors:** Alexandra Jamieson, Lamia Al Saikhan, Betty Raman, Lamis Alghamdi, Nathan J Cheetham, Pauline Conde, Richard Dobson, Alba Fernández-Sanlés, Amos Folarin, Lucy J Goudswaard, Lee Hamill Howes, Siana Jones, Stefan Neubauer, Michele Orini, Iain Pierce, Yatharth Ranjan, Alicja Rapala, Stephen M Smith, Carole Sudre, Ellen J Thompson, James Wild, Dylan Williams, Andrew Wong, Claire J Steves, Nic Timpson, Nishi Chaturvedi, Alun Hughes

**Affiliations:** 1 Department of Population Science & Experimental Medicine, University College London, London, UK; 2 MRC Unit for Lifelong Health & Ageing at UCL, University College London, London, UK; 3 Department of Cardiac Technology, Imam Abdulrahman Bin Faisal University, Dammam, Saudi Arabia; 4 Radcliffe Department of Medicine, University of Oxford, Oxfordshire, UK; 5 Department of Twin Research and Genetic Epidemiology, King’s College London, London, UK; 6 Institute of Psychiatry, Psychology and Neuroscience, King’s College London, London, UK; 7 Population Health Sciences, University of Bristol, Bristol, UK; 8 MRC Integrative Epidemiology Unit, University of Bristol, Bristol, UK; 9 School of Biomedical Engineering & Imaging Sciences, King’s College London, London, UK; 10 Oxford Centre for Functional MRI of the Brain, Wellcome Centre for Integrative Neuroimaging, Nuffield Department of Clinical Neurosciences, University of Oxford, Oxford, UK; 11 Centre for Medical Image computing, University College London, London, UK; 12 School of Psychology, University of Sussex, Brighton, UK; 13 Royal Hallamshire Hospital, Sheffield, UK; 14 Guy's and St Thomas’ NHS Foundation Trust, London, UK

**Keywords:** COVID-19, Post-Acute COVID-19 Syndrome, EPIDEMIOLOGY

## Abstract

**Abstract:**

**Purpose:**

The pathogenesis of the long-lasting symptoms which can follow an infection with the SARS-CoV-2 virus (‘long covid’) is not fully understood. The ‘COroNaVirus post-Acute Long-term EffectS: Constructing an evidENCE base’ (CONVALESCENCE) study was established as part of the Longitudinal Health and Wellbeing COVID-19 UK National Core Study. We performed a deep phenotyping case-control study nested within two cohorts (the Avon Longitudinal Study of Parents and Children and TwinsUK) as part of CONVALESCENCE.

**Participants:**

From September 2021 to May 2023, 349 participants attended the CONVALESCENCE deep phenotyping clinic at University College London. Four categories of participants were recruited: cases of long covid (*long covid(+*)/*SARS-CoV-2(+*)), alongside three control groups: those with neither long covid symptoms nor evidence of prior COVID-19 (*long covid(-*)/*SARS-CoV-2(-); control group 1),* those who self-reported COVID-19 and had evidence of SARS-CoV-2 infection, but did not report long covid (*long covid(-)/SARS-CoV-2(+); control group 2*) and those who self-reported persistent symptoms attributable to COVID-19 but no evidence of SARS-CoV-2 infection (*long covid(+)/SARS-CoV-2(-); control group 3*). Remote wearable measurements were performed up until February 2024.

**Findings to date:**

This cohort profile describes the baseline characteristics of the CONVALESCENCE cohort. Of the 349 participants, 141 (53±15 years old; 21 (15%) men) were cases, 89 (55±16 years old; 11 (12%) men) were in control group 1, 75 (49±15 years old; 25 (33%) men) were in control group 2 and 44 (55±16 years old; 9 (21%) men) were in control group 3.

**Future plans:**

The study aims to use a multiorgan score calculated as the cumulative total for each of nine domains (ie, lung, vascular, heart, kidney, brain, autonomic function, muscle strength, exercise capacity and physical performance). The availability of data preceding acute COVID-19 infection in cohorts may help identify the consequences of infection independent of pre-existing subclinical disease and also provide evidence of determinants that influence the development of long covid.

**STRENGTHS AND LIMITATIONS OF THIS STUDY**

- Cohorts offer detailed prepandemic data on participants across the life course which may provide valuable insights into the aetiology of long covid and its possible long-term sequelae.
- The study was designed to harmonise with other relevant national clinical studies (eg, the Post-hospitalisation COVID-19 study and the Capturing Multiorgan Effects of COVID-19 study).
- Comprehensive and detailed multiorgan clinical measurements and longitudinal wearable measurements have been made.
- Despite study participants being recruited from population-based longitudinal studies, volunteers were unlikely to be representative of the UK population.
- During the early phase of recruitment, most participants were likely to be unimmunised. The study was not designed to investigate whether immunisation status modifies long covid.

## Introduction

 As of April 2025, there have been more than 777 million global confirmed cases of COVID-19 due to the novel coronavirus SARS-CoV-2.^1^ COVID-19 is a complex systemic disease; the acute manifestation of the infection is typically characterised by respiratory symptoms^2^ and can involve the cardiovascular, neurologic, haematologic, gastrointestinal and nervous systems.^3 4^ Studies to date have shown that the circulation, the brain and the kidneys may be vulnerable to injury during the initial phase of illness,^2 5 6^ possibly as a consequence of direct cellular toxicity, endothelial damage or a hyperinflammatory response to SARS-CoV-2 infection.^7^,^9^ Acute COVID-19 infection may lead to persistent lung,^10 11^ cardiovascular^12^ or neurological^13^ damage.

In addition to the acute consequences of COVID-19, there is growing recognition of the long-term sequelae of this infection. A plethora of symptoms including fatigue, exercise intolerance, cognitive impairment and others^14 15^ can persist for months or more after the acute phase of infection despite recovery of pulmonary function.^16^ This condition has been variously termed post-COVID-19 condition, postacute sequelae of COVID-19 or long covid. The National Institute for Health and Care Excellence (NICE) post-COVID-19 syndrome guidelines define ongoing symptomatic COVID-19 and post-COVID-19 syndrome as one or more symptoms in association and consistent with COVID-19 that continue for 4 weeks up to 12 weeks or >12 weeks with no alternative diagnosis, respectively.^17^ Long covid refers to ongoing symptomatic COVID-19 and post-COVID-19 syndrome.^17^ The cumulative global incidence of long covid is around 400 million individuals,^18^ and prevalence estimates of long covid range from ~5% in highly selected volunteers to 87% in hospitalised patients 2 months post symptom onset.^19 20^ However, more than 90% of individuals with long covid have mild acute COVID-19 and do not require hospitalisation,^21^ and we have limited understanding of the long-term implications of long covid in the general population. Therefore, population-based studies are required to understand the determinants and consequences of long covid in the wider community, which are currently unknown.

The UK is well-placed globally to address some of these questions due to the richness of its population cohort studies and the availability of universal healthcare free at the point of use. Cohorts offer detailed prepandemic data on participants across the life course. The availability of data preceding acute SARS-CoV-2 infection may help identify the consequences of infection independent of pre-existing subclinical disease and also provide evidence of determinants that influence the development of long covid.

Convalescence can be defined as the gradual recovery of health and strength after illness. The ‘COroNaVirus post-Acute Long-term EffectS: Constructing an evidENCE base’ (CONVALESCENCE) study was established as part of the Longitudinal Health and Wellbeing COVID-19 UK National Core Study to address several questions including: how should we define and diagnose sub-phenotypes of long covid in the general population? What are the risk factors for long covid and its sub-phenotypes? What are the long-term health (physical and mental) and socioeconomic consequences of having long covid? What factors enhance recovery? What might be the long-term implications of long covid for future health?

To answer some of those questions, we aimed to capture detailed and early measures of target organ abnormalities and functional status. For this purpose, we performed a deep phenotyping case-control study nested within two population-based cohorts. Here we describe its research protocol.

## Cohort description

### Participants

Participants were recruited from two population-based cohorts participating in the CONVALESCENCE study, the Avon Longitudinal Study of Parents and Children (ALSPAC) and TwinsUK. Further details of these cohorts are provided below. They were chosen as they possess prepandemic data on multisystem function (eg, exercise capacity, lung function, cognition, target organ imaging and inflammatory and cardiometabolic biomarkers) and cover a wide age range (18–101 years old). Eligible participants were identified from questionnaire responses to COVID-19 history, deployed in April 2020. Participants who had consented to being contacted about taking part in future studies were invited to the CONVALESCENCE deep phenotyping clinic.

Four categories of participants were recruited: long covid cases and three control groups (participants who did not have evidence of confirmed long covid):

- Cases: individuals with self-reporting symptoms attributable to COVID-19 of duration ≥4 weeks from disease onset and evidence of SARS-CoV-2 infection based on criteria outlined below (*long covid(+)/SARS-CoV-2(+*)).
- Control group 1: those with neither long covid symptoms nor evidence of prior COVID-19 (ie, no self-reported or test-confirmed SARS-CoV-2 infection at the time of recruitment) (*long COVID(-)/SARS-CoV-2 (-*)).
- Control group 2: those who self-reported COVID-19 and had evidence of SARS-CoV-2 infection, but did not report long covid (*long covid(-)/SARS-CoV-2(+*)).
- Control group 3: those who self-reported persistent symptoms attributable to COVID-19 but no evidence of SARS-CoV-2 infection (*long covid(+)/SARS-CoV-2(-*)).

In TwinsUK, participants were recruited in pairs from the same family, to permit future twin-pair analyses. Evidence of SARS-CoV-2 infection was defined as either self-reported positive SARS-CoV-2 PCR test or self-reported suspected SARS-CoV-2 infection with later evidence of natural infection from consideration of antibody testing and vaccination status. In addition, a valid self-reported infection or symptom start date was required to be considered SARS-CoV-2 positive.

Antibody testing based on serostatus for antinucleocapsid immunoglobulin G (anti-N IgG) and antispike IgG (anti-S IgG) was performed using commercial kits (Thriva Solutions, UK). Initial assays were performed on finger-prick blood samples collected via posted kits.^22^ Assays were subsequently repeated on venous blood samples taken at the time of the deep phenotyping clinics. Results were interpreted based on Centers for Disease Control and Prevention Interim Guidelines for COVID-19 Antibody Testing in Clinical and Public Health Settings (https://archive.cdc.gov/#/results?q=https://www.cdc.gov/coronavirus/2019-ncov/hcp/testing/antibody-tests-guidelines.html&start=0&rows=10).

Long covid (ie, ongoing symptomatic COVID-19 and post-COVID-19 syndrome) status was defined based on the NICE post-COVID-19 syndrome guidelines: one or more symptoms in association and consistent with COVID-19 that continue for 4 weeks up to 12 weeks or >12 weeks with no alternative diagnosis.^17^ Symptoms were recorded at the time of clinic attendance to distinguish people with ongoing or resolved long covid. To be defined as having had long covid, individuals did not need to be symptomatic at the time of clinic attendance.

### Avon Longitudinal Study of Parents and Children

ALSPAC is a multigenerational, population-based prospective birth cohort study established in the early ‘90s. All pregnant women resident in Avon, UK with an expected date of delivery between 1 April 1991 and 31 December 1992 were invited to take part in the study. 13 988 children from 14 541 pregnancies were originally enrolled, and further recruitment phases included more eligible participants in following years. Mothers, partners and children from this recruitment have been followed up at multiple time points. The study website (http://www.bristol.ac.uk/alspac/researchers/our-data/) contains details of all available data through a fully searchable data dictionary and variable search tool. Data have also been collected on 12 113 partners of the pregnant women who have been invited to take part since 2010.^23 24^

In April 2020, ALSPAC participants were first invited to complete a series of questionnaires focused on COVID-19. A serology study was carried out in April 2021, in which SARS-CoV-2 infection status was defined based on measures of anti-N IgG and anti-S IgG. Participants from ALSPAC were recruited from those who responded positively (n=6988) to the following question in the fifth COVID-19 questionnaire (July–October 2021): ‘Would you be happy to be contacted about taking part in future studies, which may involve a trip to London (this applies regardless of whether or not you have had COVID-19)?’. Between July and December 2021, participants were asked to report the duration of their COVID-19 symptoms (less than 2 weeks, 2–3 weeks, 4–12 weeks, more than 12 weeks).

### TwinsUK

The TwinsUK registry, established in 1992, consists of a volunteer sample of twins born in England, Scotland, Northern Ireland or Wales. As of March 2024, the sample consisted of 14 575 adult twins (55% monozygotic and 43% dizygotic) who were between 18 years and 101 years of age. Most of the twins are women.

Between May 2020 and April 2021, twins provided samples for antigen and antibody testing over multiple time points. From this data, evidence of natural SARS-CoV-2 infection was assessed. A classification of a ‘natural infection status’ was derived from the interpretation of results of laboratory antigen tests where available and anti-N IgG and anti-S IgG tests, accounting for the antibody response due to vaccination (vaccination dates provided from self-reported questionnaires).

### Aims

The key hypotheses for the CONVALESCENCE deep phenotyping study are that:

1. Long covid may be associated with multiorgan abnormalities.
2. Multiorgan abnormalities may be associated with the pattern of symptoms of long covid.
3. Prepandemic characteristics may predict susceptibility to long covid and its sequelae.
4. Long covid may be associated with impaired exercise tolerance, disturbance of physiological function and poorer self-reported physical health, mental health and cognitive function over a period of months assessed out of clinic using a wearable device and a smartphone app.

### Eligibility criteria

Participants were considered eligible for enrolment into the study if they fulfilled all of the inclusion criteria and none of the exclusion criteria as defined below.

### Inclusion criteria

Inclusion criteria for participants in the deep phenotyping study included:

- Ability to provide written informed consent and willingness to share incidental findings with their general practitioner (GP).
- Having serological evidence about SARS-CoV-2 infection status.

### Exclusion criteria

Exclusion criteria in the deep phenotyping study were:

- Inability or unwillingness to attend the clinical assessment.
- Terminal illness, severe comorbidities or contraindicating factors preventing participation.
- Pregnancy.

### Analysis plan and sample size

The primary outcome for hypothesis 1 (multiorgan abnormalities) was assessed using a multiorgan score calculated as the cumulative total for each of nine domains (ie, lung, vascular, heart, kidney, brain, autonomic function, muscle strength, exercise capacity and physical performance). Abnormalities in each domain were scored as no abnormalities (0) or mild (1), moderate (2) or severe (3). Scores were derived using predefined clinical or literature cut-offs where possible or data-driven approaches where these were unavailable. Age, sex, occupational social class, educational attainment, smoking status, frequency of habitual physical activity, comorbidities, number of SARS-CoV-2 infections, vaccination status, time between the clinic and the first SARS-CoV-2 infection and twin status were used as covariates.

At the time of initial study design, we hoped to recruit 800 people to CONVALESCENCE deep phenotyping; however, early pilot studies indicated that this target was not feasible due to the increasing ubiquity of SARS-CoV-2 infection and its impact on recruitment, and thus, on the projected duration of the study. We therefore revised the sample size calculations based on estimates of the likely prevalence of multiorgan abnormalities based on newly available data from healthy controls participating in the Capturing Multiorgan Effects of COVID-19 (C-MORE) study.^25 26^

For hypothesis 1 (multiorgan abnormalities), we planned to use generalised linear models comparing long covid with pooled controls (assuming 1:1 case vs control). We assumed that covariates would contribute no more than a combined r^2^=0.2 and neglected possible shrinkage in variance due to inclusion of twins. Under these assumptions, a sample size of 350 would be sufficient to detect a clinically important standardised difference in the multiorgan score ≥0.3 between cases and controls at alpha=0.05 with 80% power. We planned to use multiple imputation to account for missing data under an assumption of missing at random.

### Data handling and management

**Figure 1:**
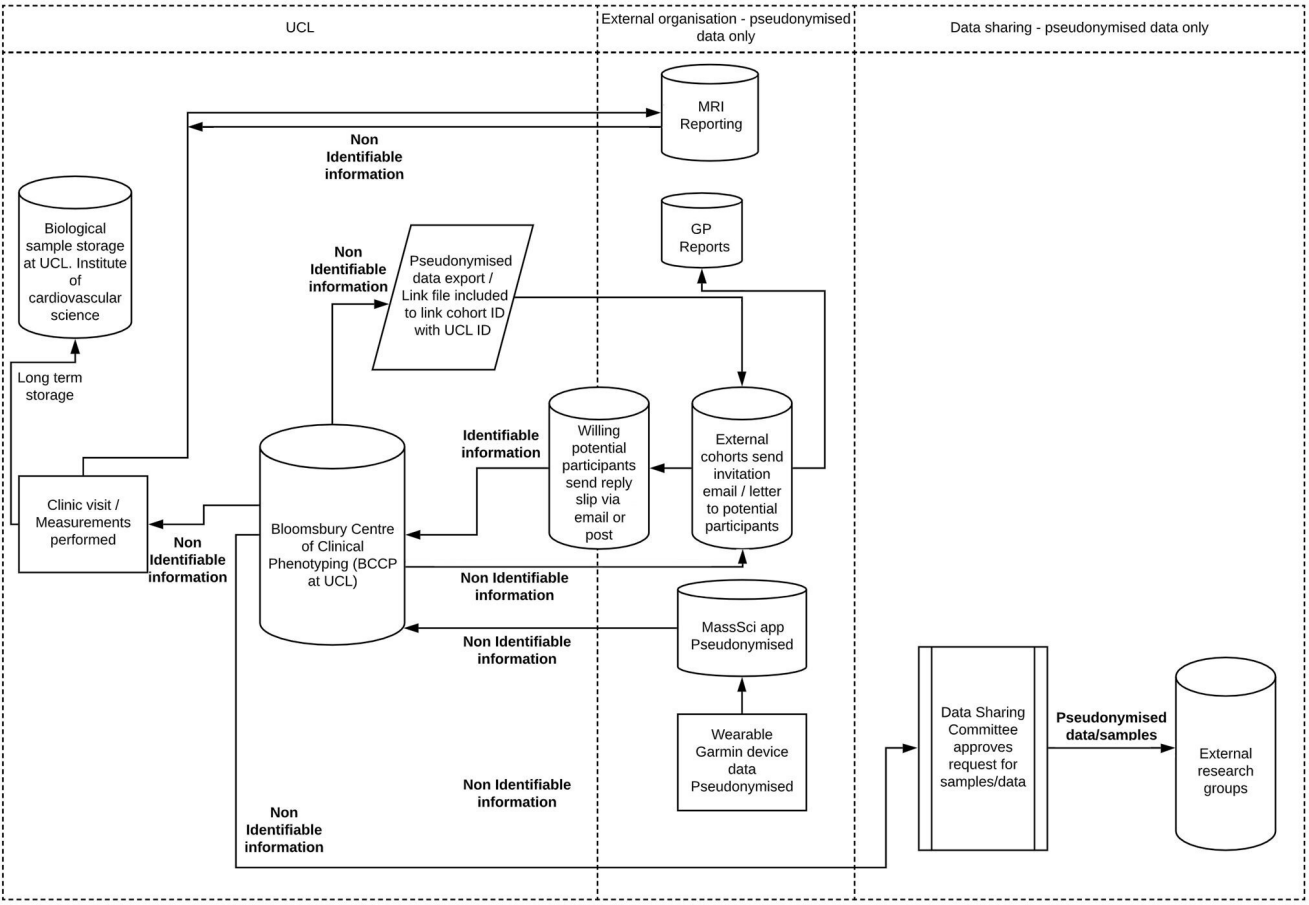
CONVALESCENCE Data Flow Chart. GP, general practitioner; UCL, University College London

All data were pseudonymised and did not contain personally identifiable information. All clinical data collected during the study were captured via direct entry or imported into a secure electronic case report form using REDCap (Research Electronic Data Capture).^27^ Data were stored in the University College London (UCL) Data Safe Haven (UCL trusted research environment). A data flow chart for the CONVALESCENCE study is summarised in figure 1.

### Patient and public involvement and engagement

Both cohorts had established patient and Public Involvement and Engagement and public involvement and engagement processes and advisory groups who contributed to the design of questionnaires, research plans and participant information sheets. Representatives met in 2021 to discuss and make contributions to CONVALESCENCE study plans and subsequently met regularly and provided advice.

### Investigations

Participants were invited to the Bloomsbury Centre for Clinical Phenotyping (BCCP) at UCL (UK) for investigations lasting between 3 hours and 4 hours between September 2021 and May 2023. Before clinic attendance, all participants completed a questionnaire about their demographics, health, physical activity and functional capacity. A telephone call was made to participants 24 to 48 hours prior to their scheduled clinic visit to ensure that new onset (within the past 14 days) acute COVID-19 symptoms were not present. During a face-to-face interview at the time of clinic attendance, information relating to safety, relevant current health, medical history and current medications was recorded.

### Anthropometric measurements and body composition

Height (cm) was measured using a stadiometer (SECA 217, Hamburg, Germany). Weight (kg) and an estimate of body composition including body fat (%) and fat mass (kg) were measured using bioimpedance scales (BC-418 or MC-780MA, Tanita, Japan). Body Mass Index (BMI) was derived from these measures.

### International Physical Activity Questionnaire

Participants completed the International Physical Activity Questionnaire – Short Form (IPAQ- SF). The IPAQ assesses both the types and intensity of physical activity that individuals perform as part of their daily lives in addition to the amount of time spent sitting in the last 7 days.^28^

### Blood and urine sample collection

Point-of-care testing was performed to measure creatinine (StatSensor Xpress, Nova Biomedical, USA) and haemoglobin (Hb 801, HemoCue, Sweden). Non-fasting blood samples (maximum of 30 mL) and a urine sample (10 mL) were collected from all willing participants. Three 4.0 mL EDTA and three clotted 3.5 mL serum-separating tube whole blood samples were collected. These were processed to provide one whole blood EDTA sample, two plasma EDTA samples and three serum samples. 500 µl samples were aliquoted into polypropylene cryovials and stored at −80°C. A random urine sample was collected in a polypropylene universal container by the participant, and later aliquoted into 10 mL slender conical tubes for storage at −80°C. These samples will be available for future studies (which may include biomarker measures, multiomic analyses and future stem cell extraction).

### Multiorgan MRI

The MRI protocol was designed to assess structure and function of the lungs, brain, heart, liver, cardiovascular system and kidneys and aligned with a previous national MRI follow-up study, C-MORE.^25^ This involved a multiorgan scan (≤65 min) using a 3-Tesla Siemens Prisma scanner (table 1). Lung MRI data include free breathing T1- and T2-weighted sequences to qualitatively and semiquantitatively assess the extent of lung parenchymal involvement and perfusion imaging to assess lung perfusion. Brain MRI data include T1- and T2-weighted imaging to assess inflammation, diffusion-weighted imaging to assess ischaemic injury, susceptibility-weighted imaging to assess possible haemorrhage and arterial spin labelling to quantitatively assess cerebral blood flow. Abdominal MRI data include liver T1 mapping to assess liver fibrosis and inflammation, a multiecho gradient echo to assess liver fat and iron, a kidney T1 map to assess fibrosis and inflammation and an R2* map to assess renal oxygenation. Cardiac MRI data include cine steady-state free precession imaging to assess biventricular volumes, mass and function, T1 and T2 mapping for assessment of myocardial inflammation and fibrosis, late gadolinium enhancement imaging (bolus dose of gadolinium-based contrast) to assess focal fibrosis and aortic distensibility. 15 min after contrast injection, slice-matched postcontrast T1 mapping was undertaken to assess extracellular volume for estimation of diffuse fibrosis.

**Table 1:** Table of sequences used and typical sequence parameters

	Sequence	Typical sequence parameters
Brain	High-resolution T1-weighted magnetisation prepared rapid gradient echo	TR/TE/TI 2000/1.97/880 ms. Acceleration factor 2. Flip angle 8°. FOV 256 mm. FOV phase 100%. Matrix 256×256×208. Voxel dimension 1×1×1 mm.
	Fluid-attenuated inversion recovery(T2-FLAIR-SPACE)	TR/TE/TI 5000/386/1800 ms. Acceleration factor 3. FOV 256 mm. FOV phase 100%. Matrix 256×256×192. Voxel dimension 1×1×1.05 mm.
	Diffusion-weighted imaging	TR/TE 10700/78 ms. Flip angle 90°. FOV 230 mm. FOV phase 100%. Matrix 104×104×72. Voxel dimension 2×2×2 mm. 6/8 Partial Fourier, b=0, 1000 s/mm2, 3 sequentially applied diffusion gradient directions (x, y, z), plus blip-reversed b=0*
	Susceptibility-weighted imaging (3D-SWI)	TR/TE_1_/TE_2_ 27/9.42/20 ms. Acceleration factor 2. Flip angle 15°. FOV 230 mm. FOV phase 91%. Matrix 232×256×48. Voxel dimension 0.9×0.9×3 mm.
Liver	Liver multiscan (LMS)magnitude only thin-slice T2* (MOST)	TR/TE/Echo spacing 12/1.3–9.84/1.2 ms. Flip angle 9°. FOV 440 mm. FOV phase 81%. Matrix 256×208. Slice thickness 3 mm.
	LMS modified look-locker inversion recovery (MOLLI)T1 native and post-contrast map	TR/TE/initial TI 373/1.05/100 ms. Flip angle 35°. FOV 440 mm. FOV phase 75%. Matrix 382×288. Voxel dimension 2.3×2.30.8 mm. Slice thickness 8 mm. Distance factor 25%.
	LMS iterative decomposition of water and fat with echo asymmetry and least-squares estimation	TR/TE/Echo spacing 15/1.1–13.2/1.1 ms. Flip angle 3°. FOV 440 mm. FOV phase 91%. Matrix 256×232. Slice thickness 10 mm. Distance factor 50%.
Lungs	Free breathing thoracic axial half-fourier single-shot turbo spin echo	TR/TE 750/49 ms. Flip angle 160°. FOV 380 mm. FOV phase 72%. Matrix 256×184. Slice thickness 8 mm. Distance factor 0%. 35 slices.
	Inspiratory and expiratory radio-frequency spoiled 3D coronal gradient echo (GRE) native and post-contrast	TR/TE 1.87/0.67 ms. Flip angle 3°. FOV 400 mm. FOV phase 100%. Matrix 128×128. Voxel dimension 3.1×3.1×3 mm. Slice thickness 3 mm, Distance factor 20%. 88 slices.
	Lung and kidney perfusion – dynamic contrast enhanced GREAdministration of 0.05 mmol/kg GBCA	TR/TE 1.47/0.5 ms. Flip angle 17°. FOV 450 mm. FOV phase 100%. Matrix 64×64. Voxel dimension 7×7×6.3 mm. Slice thickness 6.3 mm. Distance factor 20%. 40 slices/slab. 60 measurements.
Kidneys	Multi echo GRE oblique coronal T2*	TR/TE/Echo spacing 81/9.84–63.96/4.92 ms. GRAPPA factor 3. Flip angle 25° FOV 288 mm. FOV phase 100%. Matrix 192×192. Voxel dimension 1.5×1.5×5 mm. Slice thickness 5 mm.
	MOLLI T1 oblique coronal map	TR/TE/initial TI 274.3/1.15/100 ms. Flip angle 35°. FOV 320 mm. FOV phase 100%. Matrix 384×384. Voxel dimension 0.8×0.8×5.5 mm. Slice thickness 5.5 mm.
	Long axis (LAX) cines: 4, 2, 3-chamber.	TR/TE/Echo spacing 37.92/1.38/3.16 ms. Flip angle 65°. FOV 380 mm. FOV phase 84%. Matrix 208×174. Slice thickness 7 mm. Distance factor 20%.
Heart	Native and post-contrast T1 mapping (Shortened Modified-Look-Locker Inversion Recovery): Basal, mid and apical left ventricle (LV) short axis (SAX).	TR/TE/initial TI 379/1.07/100 ms. Flip angle 35°. FOV 360 mm. FOV phase 75%. Matrix 288×384. Slice thickness 8 mm. Distance factor 25%.
	Native T2 mapping: Basal, mid and apical LV SAX.	TR/TE/Echo spacing 222/1.3/3 ms. Flip angle 20°. FOV 360 mm. FOV phase 80%. Matrix 154×192. Slice thickness 8 mm. Distance factor 25%.
	Aortic distensibility: level of the right pulmonary artery and diaphragm.	TR/TE 43.5/1.26 ms. Flip angle 55°. FOV 380 mm. FOV phase 100%. Matrix 384×384. Slice thickness 6 mm. Distance factor 20%. 100 phases acquired.
	SAX cine stack.	TR/TE 37.92/1.38 ms. Flip angle 65°. FOV 380 mm. FOV phase 84%. Matrix 208×174. Slice thickness 7 mm. Distance factor 43%.
	Inversion scout: 4-chamber LAX view.	TR/TE 28.71/1.41 ms. Flip angle 35°. FOV 340 mm. FOV phase 81.4%. Matrix 192×78. Slice thickness 8 mm. Distance factor 20%.
	Late gadolinium enhancement: 4, 2, 3-chamber LAX and contiguous stack of LV SAX images.	TR/TE/Echo spacing 700/1.19/2.90 ms. Flip angle 55°. FOV 380 mm. FOV phase 68.8%. Matrix 256×176 Slice thickness 7 mm. Distance factor 43%.

FOV, Field-of-view; GBCA, Gadolinium-based contrast agent; GRAPPA, Generalized Autocalibrating Partially Parallel Acquisitions; TE, Echo time; TI, Inversion time; TR, Repetition time.

### Lung function measurements

Spirometry was used to assess lung function. Three measurements were taken using an Easy-On-PC TrueFlow Spirometer (NDD Medical Technologies, France), with the highest reading recorded. Forced expiratory volume in one second (FEV_1_), forced vital capacity (FVC) and FEV_1_/FVC Z-scores were calculated using the Global Lung Function Initiative reference equations.^29 30^

### Cardiovascular measurements

A BP+device (USCOM, Australia) was used to measure brachial blood pressure (BP), central BP and pulse rate in duplicate in the left arm. If the left arm could not be used, the right arm was used. Measurements were taken in three positions with an appropriately sized cuff: first, three consecutive measurements in a resting seated position. Participants were then asked to lie supine for at least 2 min and three consecutive supine measurements were taken. Finally, participants were asked to stand from the supine position, and two measurements were taken at 1 min and 3 min post standing. The change in brachial BP, central BP and pulse rate was calculated at both time points post standing. BP waveforms were stored as xml files for future analysis.

A standard 12-lead ECG was performed at rest in the supine position using a CardioPerfect device (Welch Allyn, New York, USA). An additional 5-minute recording of the 12-lead ECG was then performed to assess resting heart rate variability.

### Retinal optical coherence tomography angiography

Optical coherence tomography a (OCT) angiography imaging was performed to assess the retina and the retinal vasculature. This included DRI OCT Triton Imaging (Topcon IMAGEnet6 software) of both eyes:

1. Fundus photography with fluorescence turned off.
2. OCT of the macula, 3D scan.
3. OCT of the optic nerve, 3D scan (disc).
4. OCT angiography of the macula (4.5×4.5 mm).

Novel machine learning techniques were used to analyse raw image files post data collection; the measures obtained are shown in table 2.

**Table 2:** Optical coherence tomography measures

Colour fundus photography	Optical coherence tomography
Disc height	Inner limiting membrane
Disc width	Retinal nerve fibre layer
Cup height	Ganglion cell layer
Cup width	Inner plexiform layer
Fractal dimension	Retinal Pigment epithelium
Vessel density	Bruch’s membrane
Distance tortuosity	Inner nuclear layer
Squared curvature tortuosity	External limiting membrane
Tortuosity density	
Artery fractal dimension	
Artery vessel density	
Artery average width	
Artery distance tortuosity	
Artery squared curvature tortuosity	
Artery tortuosity density	
Vein fractal dimension	
Vein vessel density	
Vein average width	
Vein distance tortuosity	
Vein squared curvature tortuosity	
Vein tortuosity density	

### Exercise capacity and cardiopulmonary fitness

All exercise tests were conducted according to the ATS/ACCP (2003) guidelines for cardiopulmonary exercise testing (CPET).^31^ Participants were not invited to exercise if they met any of the absolute contraindications given in the guidelines.

CPET was performed using a semirecumbent cycle ergometer (Ergoline 900, Germany) and metabolic cart (Quark Cosmed, Italy).^32^ Following 1 min of rest and 2 min of warm-up cycling at 5 Watts, the work rate was incrementally increased using a ramp protocol in an individualised manner by either 15 Watts, 20 Watts, 25 Watts or 30 Watts each minute based on the Wasserman weight algorithm.^33^ The test was terminated if the participant (1) reached their age-predicted (220-age) maximum heart rate (2) experienced limiting symptoms or (3) developed arrhythmia, hypotension (systolic BP drop of >10 mm Hg despite increasing workload) or (4) an excessive BP rise during the test (systolic BP >250 mm Hg). Expired gases were analysed breath-by-breath, and heart rate and rhythm were measured using a continuous 6-lead ECG (Quark CPET, Cosmed, Italy).

Expired gases, ventilation and heart rate were monitored continuously throughout the test. Data were smoothed using a rolling 30-second average where necessary. Oxygen saturation of peripheral arterial blood (SpO_2_, either finger or forehead probe) was monitored continuously during CPET, and BP was measured every 3 min throughout exercise and recovery using a motion insensitive device (Tango M2, Suntech Medical, North Carolina, USA) according to guidelines.^31^ Capillary lactate was measured prior to exercise and again at peak effort using a point-of-care lactate analyser (Nova StatStrip Xpress, Nova Biomedical, UK). Participants were asked to score both breathlessness (dyspnoea) and leg fatigue using the Borg CR10 scale on termination of exercise.

Key CPET outcome measures derived included: peak oxygen consumption (peak VO_2_), peak heart rate, oxygen uptake efficiency slope, peak O_2_ pulse, ventilatory equivalent of carbon dioxide (VE:VCO_2_) slope and ratio of oxygen uptake to work rate (VO_2_/Work Rate). Maximum predicted VO_2_ was estimated using the equations of Wasserman and Whipp for men and women.^34^ Anaerobic threshold and respiratory compensation point were determined using the V-slope method and via visual inspection of the change in ventilatory equivalents and end-tidal partial pressure of CO_2_ during incremental exercise.

### Exercise echocardiography

A focused stress echocardiographic examination was performed during the CPET. The scan was performed using a standardised protocol on a Philips EPIQ 7G scanner equipped with an X5-1 transducer in accordance with the American Society of Echocardiography guidelines.^35^,^37^ A 3-lead ECG was recorded ensuring clear QRS complexes throughout the examination. The stress echo protocol consisted of four phases as follows: baseline (at rest), low intensity (at the warm-up stage of the CPET; 5 Watts for 2 min), intermediate intensity (at 65±5% of age-predicted maximum heart rate) and recovery (at 3 min post exercise). The following images obtained from the apical 4-chamber window were acquired at each stage: (1) left ventricular (LV) 3D imaging, (2) tissue pulsed wave (PW) Doppler imaging over the lateral mitral valve (MV) annulus and (3) tissue PW Doppler imaging over the right ventricular free wall. During the acquisition of baseline images, settings were optimised including depth, sector width, gain, scale and baseline from which the system saved and maintained image optimisation settings for subsequent phases. Optimal images were obtained prior to recording, and five cardiac cycles were recorded.

For 3D imaging, a 1-beat full-volume LV 3D dataset was obtained, and depth and sector width settings were adjusted as needed for maximum frame rate. Harmonic imaging and HPen were used for optimal image spatial resolution. The 3D image acquisition mode of the system was adjusted to ensure optimal temporal resolution while preserving adequate spatial resolution. If the quality of the loop was unacceptable, it was rejected, and the acquisition was repeated. Once baseline images were acquired, participants were asked to start cycling and imaging was repeated at the predefined time points.

Data were routinely visually inspected for adherence to the protocol and clarity of the images. Images were stored on the internal drive of the ultrasound machine and were then transferred to the local server for archiving and offline analyses.

#### 3D speckle tracking echocardiography analysis

Analysis was performed off-line using Tomtec (TTA2.51.02, TomTec Image Arena, TomTec Imaging Systems, Munich, Germany) by a single trained reader, with minimal manual adjustments to the endocardial border. Image quality was assessed based on a five-point score as previously described^38^ and summarised in table 3.

**Table 3:** 3D echocardiography image quality grading

Grade	Definition
Good (score 1)	Clear visualisation of endocardium in all 16 segments.
Fair (score 2)	Unclear visualisation of endocardium in ≤2 segments or presence of minor artefacts, for example, apical noise.
Adequate (score 3)	Unclear visualisation of endocardium in ≤6 segments.
Poor (score 4)	Unclear visualisation of endocardium in >6 segments, but reliable tracking throughout the cardiac cycle using the adjacent segments as a reference.
Unacceptable (score 5)	Unacceptable visualisation of the LV endocardial boundaries, or ≥4 segments being outside the image sector.

LV, left ventricle.

Following the IQ assessment of each image, the optimal heartbeat from the acquired loop was selected and the automated placement of the LV apex, MV and aortic valve was adjusted as required. Automated tracing of the endocardium during both systolic and diastolic phases by the software minimised the requirement for manual adjustments of the endocardial border across various views including short axis, apical 4-chamber, 3-chamber and 2-chamber views. Key parameters included in the analyses were 3D speckle tracking echocardiography-derived indices of 3D global longitudinal strain and 3D ejection fraction. These indices were also assessed across the four phases of the imaging protocol as described above. LV mass was computed automatically in 3D, with manual adjustments made when deemed necessary. The data and video loops were exported as text files and AVI files, respectively.

### Near infrared spectroscopy

A near infrared spectroscopy (NIRS) device (Portamon, Artinis Medical Systems, Netherlands) was positioned on the lateral portion of the gastrocnemius in the left calf. The device was secured using micropore tape and covered using a black neoprene sleeve to prevent ambient light interference with the NIRS signal. The device was set to sample at 10 Hz.

Oxygenated and deoxygenated haemoglobin and tissue saturation index (TSI) were measured continuously in the lateral gastrocnemius by continuous wave NIRS during the CPET. This non-invasive optical technique was used to examine muscle perfusion during exercise and recovery of muscle oxygenation post exercise.^39^,^41^ Analysis of NIRS data was performed in MATLAB (MathWorks Inc, Massachusetts, USA) using custom written programmes. TSI was estimated using spatially solved spectroscopy.^42^

On termination of the CPET, adipose tissue thickness was measured three times at the site of the NIRS measurement by ultrasound (Vivid I, GE, Illinois, USA). An average of the three measurements (cm) was recorded.

### Physical capability - short physical performance battery

Physical capability was assessed using the National Institute on Aging Short Physical Performance Battery (SPPB).^43^ The SPPB has been associated with self-reported disability and prediction of mortality and nursing home admission.^43^ The battery consists of four measures to assess balance, gait, strength and endurance.

The standing balance test requires the participant to stand on one leg with arms folded across the chest, for up to a maximum of 30 s. The test ends when 30 s has elapsed or when the participant loses their balance, whichever occurs sooner. This was performed for both legs once with the eyes open and once with the eyes closed.

Participant’s self-paced gait was measured over a 2.44 m (8 feet) flat distance and recorded to the closest 1/100^th^ s using a stopwatch. The test was performed a total of three times.

Hand-grip strength was measured with a hand-grip dynamometer (Takei 5401 Digital Dynamometer) which consists of a gripping handle (with a strain-gauge transducer) and an amplifier with digital displays. Hand dominance was documented, and three measurements were performed in the dominant hand.

Participants were asked to perform 10 complete chair rises (5, if 10 was not feasible) with their arms folded across their chest. The time in which it took the participant to complete the chair rises was measured to 1/100^th^ s using a stopwatch.

### Wearable Measurements

Participants were provided with a Garmin vivoactive 4S smartwatch and assisted with the installation of two accompanying mobile phone apps onto their smartphone using pseudonymised study participant ID username and passwords allocated by the study team. First, the Garmin Connect app was installed and linked to the allocated smartwatch. This enables the transfer of physiological data from the watch to the mobile phone (Garmin Connect) via Bluetooth. Garmin Connect provides the functionality for participants to view their daily, weekly and monthly summary statistics for parameters such as heart rate, step count, pulse oximetry and respiration. Second, participants were enrolled into the CONVALESCENCE Study on the Mass Science app (developed by collaborators at Kings College London).^44^ During this process, participants consent to the transfer of their physiological data from Garmin to CONVALESCENCE servers at UCL for research purposes.

In addition to facilitating data transfer and acquisition at scale, the Mass Science app hosts questionnaires, provides written and video instructions to perform two remote measures of physical capacity (chair rises test and 6-minute walk test (6MWT)) and allows participants to record the date and time in which they perform these tasks or reason for non-completion.

### Questionnaires and cognitive assessment

Study participants were asked to complete the Generalised Anxiety Disorder-7, Patient Health Questionnaire-8 and Cognitron cognitive task (https://braingames.cognitron.co.uk/), a cognitive test platform designed for remote assessment of cognition and mental health.^45^,^47^ Cognitron was hosted on the Mass Science App and undertaken on a fortnightly basis.

### Out of clinic assessments of physical capacity

The Garmin vivoactive 4S allowed for continuous recording of physiological parameters such as heart rate, step count, pulse oximetry and respiration rate, typically collected at a sampling rate of one per 5–10 s. However, the sampling rate can be increased to every second during activity test recordings. In addition to the assessment of habitual activity and heart rate, participants were asked to perform two standardised activity tests: a chair rise test and a 6MWT fortnightly for the first 6 months and quarterly thereafter. The chair rise test involved the participant undertaking 10 complete chair rises (5 if 10 is not feasible) with arms folded across their chest, followed by a 1-minute seated recovery in their free-living environment. This was recorded as a ‘Cardio’ activity on the smartwatch. The 6MWT involved the participant completing a 6-minute walk followed by a 1-minute standing recovery outside in a suitable location in their local community. This was recorded as a ‘Walk’ activity on the smartwatch. Participants were prompted to perform these activities by the Mass Science app providing reminders via mobile phone notification.

### Incidental findings

Screening policies were put in place regarding any abnormalities found incidentally during the study visit to determine whether they were clinically actionable and thus required follow-up. Participants were made aware from the participant information sheet that research scans and procedures were not for diagnostic purposes and therefore were not a substitute for a clinical appointment or investigation. Investigators gained permission from the participant to contact their GP directly so that the GP could subsequently arrange appropriate clinical case management. We aimed to have images screened within 6 weeks of everyone’s study visit date.

Brain MRI images were screened by an experienced neuroradiologist. This included any abnormalities in intracranial imaging appearances, intracranial neuroparenchymal volumes, evidence of any appearance abnormalities to the major dural venous sinuses and intracranial arteries ischaemia, an established infarct or gross haemorrhage or intracranial space-occupying lesion.

Cardiac MRI images were screened by an experienced cardiologist. Images were screened for thoracic anatomy abnormalities, cardiac function, tissue characterisation and any other relevant abnormalities.

OCT images were screened by a team at Moorfields Eye Hospital. Two ophthalmologists screened the images and flagged any complex cases for referral to a consultant ophthalmologist. Common irregularities were screened for using all collected image types.

## Findings to date

### Study participant characteristics

A total of 349 participants were recruited to this study, of which there were 141 (52±15 years old; 21 (15%) men) cases, 89 (54±15 years old; 11 (12%) men) controls in group 1, 75 (49±15 years old; 25 (33%) men) controls in group 2 and 44 (54±15 years old; 9 (21%) men) controls in group 3. table 4 describes the demographic, clinical and social characteristics of study participants.

**Table 4:** Study participant characteristics

N (%) or mean±SD	Cases	Control group 1	Control group 2	Control group 3
n	141	89	75	44
TwinsUK cohort	94 (67%)	49 (55%)	31 (41%)	12 (27%)
Age (years)	52±15	54±15	49±15	54±15
Male sex	21 (15%)	11 (12%)	25 (33%)	<10 (<23%)
Ethnicity				
White	136 (97%)	86 (97%)	73 (97%)	44 (100%)
Non-white	<10 (<7%)	<10 (<11%)	<10 (<13%)	0 (0%)
Smoking status				
Current cigarette	11 (8%)	<10 (<11%)	<10 (<13%)	<10 (<23%)
Current vaping	<10 (<7%)	<10 (<11%)	0 (0%)	0 (0%)
Ex-smoker	42 (30%)	32 (36%)	25 (33%)	16 (36%)
Never a smoker	85 (60%)	49 (55%)	45 (60%)	27 (61%)
Education				
Higher education or professional/vocational equivalents	80 (57%)	55 (62%)	44 (59%)	26 (59%)
A-levels, vocational level 3 and equivalents	38 (27%)	16 (18%)	17 (23%)	14 (32%)
GCSE/O Level grade A* to C, vocational level 2 and equivalents	20 (14%)	17 (19%)	13 (17%)	<10 (<23%)
Qualifications at level 1 and below or no qualifications	<10 (<7%)	<10 (<11%)	<10 (<13%)	<10 (<23%)
Alcohol Intake				
None	41 (29%)	25 (28%)	12 (16%)	8 (18%)
≤ 14 units per week	83 (59%)	56 (63%)	52 (69%)	29 (66%)
> 14 units per week	17 (12%)	<10 (<11%)	11 (15%)	<10 (<23%)
Cardiovascular disease	<10 (<7%)	<10 (<11%)	<10 (<13%)	<10 (<23%)
Hypertension	28 (20%)	14 (16%)	16 (21%)	<10 (<23%)
Dyslipidaemia	20 (14%)	13 (15%)	<10 (<13%)	10 (23%)
Asthma	25 (18%)	13 (15%)	10 (13%)	<10 (<23%)
Chronic obstructive pulmonary disease	<10 (<7%)	<10 (<11%)	0 (0%)	0 (0%)
Type 1 diabetes mellitus	0 (0%)	<10 (<11%)	0 (0%)	0 (0%)
Type 2 diabetes mellitus	<10 (<7%)	0 (0%)	<10 (<13%)	<10 (<23%)
Body mass index (kg/m^2^)	27.1±6.3	25.8±5.7	26.4±5.9	26.4±5.0
Waist circumference (cm)	88.4±16.8	85.9±13.2	87.8±14.6	86.7±13.4
Hip circumference (cm)	103.0±12.8	101.5±11.7	102.6±13.7	102.3±10.8
Systolic blood pressure (mm Hg)	118±16	117±15	120±13	122±17
Diastolic blood pressure (mm Hg)	73±10	71±9	74±8	74±10
Heart rate (beats per minute)	73±11	74±11	72±12	74±11

Values <10 are shown in accordance with statistical disclosure control guidelines.

97% of participants were of white ethnicity. The majority of individuals were never smokers (cases: 85 (60%); control group 1: 49 (55%); control group 2: 45 (60%) and control group 3: 27 (61%)), had received higher education or professional/vocational equivalents (cases: 80 (57%); control group 1: 55 (62%); control group 2: 44 (59%) and control group 3: 26 (59%)) and drank less than or equal to 14 units per week (cases: 83 (59%); control group 1: 56 (63%); control group 2: 52 (69%) and control group 3: 29 (66%)). Cardiovascular disease was reported in less than 10 individuals in all groups (16–23%). Hypertension was reported in 28 (20%) cases, 14 (16%) individuals in control group 1, 16 (21%) individuals in control group 2 and <10(<23%) individuals in control group 3. Asthma was reported in 25 (18%) cases, 13 (15%) individuals in control group 1, 10 (13%) individuals in control group 2 and <10 (<23%) individuals in control group 3. Chronic obstructive pulmonary disease, type 1 and type 2 diabetes mellitus were reported in either none or less than 10 individuals per group. The mean BMI was 27.1±6.3 for cases, 25.8±5.7 for control group 1, 26.4±5.9 for control group 2 and 26.4±5.0 for control group 3. The mean resting systolic BP was 118±16 mm Hg for cases, 117±15 mm Hg for control group 1, 120±13 mm Hg for control group 2 and 122±17 mm Hg for control group 3.

### Strengths and limitations

The aim of the deep phenotyping component of the CONVALESCENCE study was to elucidate the pathological basis of long covid and its long-term implications. Some previous studies have reported evidence of persistent damage following long covid, including evidence of multisystem dysfunction based on electronic health records^48^ and lung injury^49^ and heart damage^50 51^ based on imaging. However, unlike CONVALESCENCE, few of these studies were population-based and none had information about antecedent subclinical abnormalities and longitudinal characteristics.

A further strength of this study is that it was designed to harmonise with other relevant national clinical studies (eg, the Post-hospitalisation COVID-19 study^52^ and the C-MORE study^25^) to allow comparison across studies. It differs from these studies in having a population-based focus on long covid. Based on recent data, approximately one in 20 COVID-19 cases in England resulted in hospital admission (https://coronavirus.data.gov.uk/ accessed 21/01/24). It was anticipated that most, if not all, of the participants in this study would not have been admitted to hospital.

Some aspects of study design merit discussion. First, during the early phase of recruitment, most participants were unimmunised, as immunisation for COVID-19 only became available at the end of 2020 in the UK and the study was not designed to investigate whether immunisation status modifies long covid. Second, the selection of appropriate controls is a challenge for studies of long covid, and there is probably no ideal control for participants with long covid. We addressed this challenge by recruiting diverse sets of controls which should allow more insight into the consequences of long covid. We cannot rule out that long covid symptom status may have changed from the time of recruitment to clinic visit that is, the resolution of symptoms in those classified as cases or the commencement of symptoms in those classified as controls at recruitment. Control group 3 (*long covid(+)/SARS-CoV-2(-*)) is likely to mainly include participants who have experienced a different viral infection; however, given the limited testing during the early phase of the pandemic and limited sensitivity of the antibody tests, some in this group may have been infected with COVID-19. Third, while study participants were drawn from population-based longitudinal studies, volunteers were unlikely to be representative of the UK population in many respects, including socioeconomic status and lifestyle. The possibility of selection bias should be considered when interpreting the results, and this may limit the generalisability of our findings.

In conclusion, the deep phenotyping component of the CONVALESCENCE study aims to establish whether long covid is associated with multiorgan abnormalities, whether prepandemic characteristics (particularly subclinical disease) contribute to susceptibility to long covid and whether abnormalities are linked to symptoms of long covid. This should provide valuable insight into the aetiology of long covid and its possible long-term sequelae which may inform clinical practice, public health policies and future research.

## Data Availability

Data are available upon reasonable request.
