# Supplementary material for: Cohort profile: characterisation, determinants, mechanisms and consequences of the long-term effects of COVID-19 – providing the evidence base for health care services (CONVALESCENCE) in the UK
Source: BMJ Open. 2025 Jun 5;15(6):e094760. doi: 10.1136/bmjopen-2024-094760 (PMC12142084; doi:10.1136/bmjopen-2024-094760)
Supplement: Supplementary file 1 [file bmjopen-2024-094760.reviewer_comments.pdf]

PEER REVIEW HISTORY

BMJ Open publishes all reviews undertaken for accepted manuscripts. Reviewers are asked to complete a checklist review form and are provided with free text boxes to elaborate on their assessment. These free text comments are reproduced below.

ARTICLE DETAILS

Title (Provisional)

Cohort profile: Characterisation, determinants, mechanisms and consequences of the long-term effects of COVID-19: providing the evidence base for health care services (CONVALESCENCE) in the United Kingdom

Authors

Jamieson, Alexandra; Saikhan, Lamia Al; Raman, Betty; Alghamdi, Lamis; Cheetham, Nathan J.; Conde, Pauline; Dobson, Richard; Fernández-Sanlés, Alba; Folarin, Amos; Goudswaard, Lucy J.; Hamill Howes, Lee; Jones, Siana; Neubauer, Stefan; Orini, Michele; Pierce, Iain; Ranjan, Yatharth; Rapala, Alicja; Smith, Stephen M; Sudre, Carole; Thompson, Ellen J.; Wild, James; Williams, Dylan; Wong, Andrew; Steves, Claire J.; Timpson, Nic; Chaturvedi, Nishi; Hughes, A

VERSION 1 - REVIEW

**Reviewer**	**1**
**Name**	**Boix, Vicente**
**Affiliation**	**Alicante General University Hospital**
**Date**	**19-Jan-2025**
**COI**	**None**

None

**Reviewer**	**2**
**Name**	**Salci, Maria**
**Affiliation**	**Universidade Estadual de Maringá, Nursing Department**
**Date**	**11-Mar-2025**
**COI**	**None**

Título: Cohort profile: Characterisation, determinants, mechanisms and consequences of the long-term effects of COVID-19: providing the evidence base for health care services (CONVALESCENCE) in the United Kingdom

OVERALL EVALUATION:

The research presents an adequate structure, with some suggestions to follow. The choice of the topic addressed is of utmost importance because knowledge about long COVID, even though widely studied, is a condition that is still not fully understood and can affect millions of people worldwide.

1 INTRODUCTION

The introduction addresses a relevant topic, but is not entirely original, as post-COVID syndrome has already been widely studied. However, the use of British cohorts may be a differentiator, which could be more emphasized. The research is well-founded in scientific evidence, but the transition between the existing literature and the gap in the study could be clearer. The research question is well defined, but could be more objective. It is suggested that the originality of the study be better highlighted, the central question be reformulated and the justification be made more fluid.

2. METHOD

The study methodology is well structured and uses robust data from pre-existing cohorts, combining subjective and objective measures to assess the impact of long COVID. However, some limitations, such as the possibility of selection bias and reliance on self-reporting, should be considered when interpreting the results.

3. RESULTS AND DISCUSSION

The study presents a detailed and robust analysis of the participants, allowing a careful assessment of the factors associated with long COVID. Despite some limitations, such as representativeness and lack of vaccination data, the authors acknowledge these issues, which strengthens the reliability of the study. Exploring in more detail the impact of differences between groups and exploring alternatives for presenting censored data could further enrich the analysis.

4. CONCLUSION

The conclusion summarizes the central objectives of the study well, highlighting the investigation of the relationship between long COVID. The study aims to provide important insights into the etiology and possible long-term consequences of long COVID, which is crucial for understanding this emerging condition. The conclusion is in line with the methodological proposal of the study, reinforcing the focus on detailed characterization of participants and analysis of multiple risk factors. It would be useful to include, even if briefly, the most relevant findings. There is no mention of how the results may impact clinical practice, public health policies, or future research. Including these considerations would strengthen the relevance of the study.

REFERENCES: Pertinent.

Some suggestions, but the article is relevant for publication.

**Reviewer**	**3**
**Name**	**Bukelo, Maryann**
**Affiliation**	**North Central Regional Health Authority, Laboratory Services**
**Date**	**20-Mar-2025**
**COI**	**None**

• Abstract: Strengths and limitations to be indicated in the end.

• Introduction to include global trends before arriving at the country of study.

• Introduction to be more verbose.

• Check the entire manuscript for grammatical errors and typos.

• Definition of convalescence to be included to benefit general readers.

• Categories mentioned in methodology to be presented in point format.

• Definition of control group to be mentioned.

• To mention clearly between long and post-COVID-19.

• Strengths and limitations should be indicated at the end of the discussion.

VERSION 1 - AUTHOR RESPONSE

Reviewer Reports:

Reviewer: 1
Dr. Vicente Boix, Alicante General University Hospital

Comments to the Author:
None

We thank Dr. Vincente Boix for taking the time to review our manuscript.

Reviewer: 2
Dr. Maria Salci, Universidade Estadual de Maringá

Comments to the Author:
Some suggestions are attached, but the article is relevant for publication.

OVERALL EVALUATION: The research presents an adequate structure, with some suggestions to follow. The choice of the topic addressed is of utmost importance because knowledge about long COVID, even though widely studied, is a condition that is still not fully understood and can affect millions of people worldwide.

We thank Dr. Maria Salci for taking the time to review our manuscript.

1 INTRODUCTION The introduction addresses a relevant topic, but is not entirely original, as post COVID syndrome has already been widely studied. However, the use of British cohorts may be a differentiator, which could be more emphasized. The research is well-founded in scientific evidence, but the transition between the existing literature and the gap in the study could be clearer. The research question is well defined, but could be more objective. It is suggested that the originality of the study be better highlighted, the central question be reformulated and the justification be made more fluid.

We thank the reviewer for their comments. We have amended the introduction in our updated manuscript to better highlight the originality of the study as follows:

'Long COVID refers to ongoing symptomatic COVID-19 and post-COVID-19 syndrome [17]. The cumulative global incidence of long COVID is around 400 million individuals [18] and prevalence estimates of long COVID range from ~5% in highly selected volunteers to 87% in hospitalised patients two months post symptom onset [19]. However, more than 90% of individuals with long COVID have mild acute COVID-19 and do not require hospitalisation [20] and we have limited understanding of the long-term implications of long COVID in the general population. Therefore, population-based studies are required to understand the determinants and consequences of long COVID in the wider community, which are currently unknown.

The UK is well-placed globally to address some of these questions due to the richness of its population cohort studies and the availability of universal healthcare free at the point of use. Cohorts offer detailed pre-pandemic data on participants across the life course. The availability of data preceding acute SARS-CoV-2 infection may help identify the consequences of infection independent of pre-existing subclinical disease and also provide evidence of determinants that influence the development of long COVID.'

2. METHOD The study methodology is well structured and uses robust data from pre-existing cohorts, combining subjective and objective measures to assess the impact of long COVID. However, some limitations, such as the possibility of selection bias and reliance on self-reporting, should be considered when interpreting the results.

We thank the reviewer for their comments. We have amended our strengths and limitations section to comment further on the possibility of selection bias as follows:

'Third, while study participants were drawn from population-based longitudinal studies, volunteers were unlikely to be representative of the UK population in many respects, including socioeconomic status and lifestyle. The possibility of selection bias should be considered when interpreting the results, and this may limit the generalisability of our findings.'

3. RESULTS AND DISCUSSION The study presents a detailed and robust analysis of the participants, allowing a careful assessment of the factors associated with long COVID. Despite some limitations, such as representativeness and lack of vaccination data, the authors acknowledge these issues, which strengthens the reliability of the study. Exploring in more detail the impact of differences between groups and exploring alternatives for presenting censored data could further enrich the analysis.

We thank the reviewer for this useful suggestion. We plan to publish the results of this study in due course and have added information to the amended manuscript relating to how researchers can apply for data access.

4. CONCLUSION The conclusion summarizes the central objectives of the study well, highlighting the investigation of the relationship between long COVID. The study aims to provide important insights into the etiology and possible long-term consequences of long COVID, which is crucial for understanding this emerging condition. The conclusion is in line with the methodological proposal of the study, reinforcing the focus on detailed characterization of participants and analysis of multiple risk factors. It would be useful to include, even if briefly, the most relevant findings. There is no mention of how the results may impact clinical practice, public health policies, or future research. Including these considerations would strengthen the relevance of the study.

We thank the reviewer for their comment. We have updated the conclusion as follows:

'In conclusion, the deep phenotyping component of the CONVALESCENCE study aims to establish whether long COVID is associated with multi-organ abnormalities, whether pre-pandemic characteristics (particularly subclinical disease) contributes to susceptibility to long COVID, and whether abnormalities are linked to symptoms of long COVID. This should provide valuable insight into the aetiology of long COVID and its possible long-term sequelae which may inform clinical practice, public health policies, and future research.'

REFERENCES: Pertinent.

Some suggestions, but the article is relevant for publication

Reviewer: 3
Dr. Maryann Bukelo, North Central Regional Health Authority

We thank Dr. Maryann Bukelo for taking the time to review our manuscript.

Comments to the Author:

Abstract: Strengths and limitations to be indicated in the end.

In line with the BMJ Open Cohort Profile guidelines, strengths and limitations of this study have been reported in the section following the Abstract.

Introduction to include global trends before arriving at the country of study.

We thank the reviewer for their helpful comment. We have updated the introduction to include global trends of long COVID as follows:

'The cumulative global incidence of long COVID is around 400 million individuals [18] and prevalence estimates of long COVID range from ~5% in highly selected volunteers to 87% in hospitalised patients two months post symptom onset [19].'

Introduction to be more verbose.

We have made improvements to the introduction in the amended manuscript.

Check the entire manuscript for grammatical errors and typos.

The manuscript has been checked for grammatical errors and typos and edits have been made where required.

Definition of convalescence to be included to benefit general readers.

We thank the reviewer for this helpful suggestion. We have included a definition of convalescence in the introduction as follows:

'Convalescence can be defined as the gradual recovery of health and strength after illness. The CONVALESCENCE study ("COroNaVirus post-Acute Long-term EffectS: Constructing an evidENCE base") was established as part of the Longitudinal Health & Wellbeing COVID-19 UK National Core Study to address several questions including: how should we define and diagnose sub-phenotypes of long COVID in the general population?; what are the risk factors for long COVID and its sub-phenotypes?; what are the long-term health (physical and mental) and socioeconomic consequences of having long COVID?; what factors enhance recovery?; what might be the long-term implications of long COVID for future health?'

Categories mentioned in methodology to be presented in point format.

We thank the reviewer for their comment and agree that the categories of participants mentioned in the methodology could have been presented in a clearer format. Participant categories are now presented in point format in the updated manuscript as follows:

'Four categories of participants were recruited; long COVID cases and three control groups (participants who did not have evidence of confirmed long COVID):

- *Cases: individuals with self-reporting symptoms attributable to COVID-19 of duration ≥4 weeks from disease onset and evidence of SARS-CoV-2 infection based on criteria outlined below (long COVID(+)/SARS-CoV-2(+)).*
- *Control group 1: those with neither long COVID symptoms nor evidence of prior COVID-19 (i.e., no self-reported or test-confirmed SARS-CoV-2 infection at the time of recruitment) (long COVID(-)/SARS-CoV-2 (-)).*
- *Control group 2: those who self-reported COVID-19 and had evidence of SARS-CoV-2 infection, but did not report long COVID (long COVID(-)/SARS-CoV-2(+)).*

- *Control group 3: those who self-reported persistent symptoms attributable to COVID-19 but no evidence of SARS-CoV-2 infection (long COVID(+)/SARS-CoV-2(-)).'*

Definition of control group to be mentioned.

We thank the reviewer for their comment. We have now included a definition of control group as follows:

'Four categories of participants were recruited; long COVID cases and three control groups (participants who did not have evidence of confirmed long COVID):

- *Cases: individuals with self-reporting symptoms attributable to COVID-19 of duration $\geq$4 weeks from disease onset and evidence of SARS-CoV-2 infection based on criteria outlined below (long COVID(+)/SARS-CoV-2(+)).*
- *Control group 1: those with neither long COVID symptoms nor evidence of prior COVID-19 (i.e., no self-reported or test-confirmed SARS-CoV-2 infection at the time of recruitment) (long COVID(-)/SARS-CoV-2 (-)).*
- *Control group 2: those who self-reported COVID-19 and had evidence of SARS-CoV-2 infection, but did not report long COVID (long COVID(-)/SARS-CoV-2(+)).*
- *Control group 3: those who self-reported persistent symptoms attributable to COVID-19 but no evidence of SARS-CoV-2 infection (long COVID(+)/SARS-CoV-2(-)).'*

To mention clearly between long and post-COVID-19.

We thank the reviewer for their comment. We have made the distinction between long COVID and post-COVID-19 clearer in our introduction as follows:

'This condition has been variously termed post-COVID-19 condition, post-acute sequelae of COVID-19 (PASC) or long COVID. The National Institute for Health and Care Excellence (NICE) post-COVID-19 syndrome guidelines define ongoing symptomatic COVID-19 and post-COVID-19 syndrome as one or more symptoms in association and consistent with COVID-19 that continue for 4 weeks up to 12 weeks or >12 weeks with no alternative diagnosis, respectively [17]. Long COVID refers to ongoing symptomatic COVID-19 and post-COVID-19 syndrome [17]. The cumulative global incidence of long COVID is around 400 million individuals [18] and prevalence estimates of long COVID range from ~5% in highly selected volunteers to 87% in hospitalised patients two months post symptom onset [19].'

Strengths and limitations should be indicated at the end of the discussion.

We thank the reviewer for their comment. In line with the BMJ Open requirements for a cohort profile, the discussion section now forms the strengths and limitations section of the manuscript. We have made this clearer in the updated manuscript.
